# Does the Addition of Locked Cross-Elements Prevent Screw Subsidence Following Locking Plate Fixation of Proximal Humerus Fractures?

**DOI:** 10.7759/cureus.114179

**Published:** 2026-08-08

**Authors:** Htoo Lu, Joshua Ong, Priyadarshi Amit, Joon Ha, Kalpesh Vaghela, Livio Dimascio

**Affiliations:** 1 Trauma and Orthopaedics, Royal London Hospital, London, GBR; 2 Orthopedics, Royal London Hospital, London, GBR; 3 Upper Limb, Royal London Hospital, London, GBR; 4 Trauma and Orthopaedic Surgery, Royal London Hospital, London, GBR

**Keywords:** cross-element, locking plate, pantera, philos, proximal humerus fracture

## Abstract

Purpose

To compare screw subsidence and screw penetration in proximal humerus fracture fixation using the PANTERA plate with cross-elements (Toby Orthopaedics, Miami, FL, USA) versus the conventional PHILOS plate (Synthes, West Chester, PA, USA).

Methods

Twenty-seven patients treated with the PANTERA plate (cross-element group) were matched by age, gender, and injury side with sixty patients treated using the PHILOS plate (conventional group). Intraoperative and post-operative radiographs were analysed for humeral head subsidence (HHS), defined as the change in corrected distance from the screw tip to the articular surface along the screw axis, measured on coronal (Cor-HHS) and axial (Ax-HHS) views.

Results

The mean patient age was 55.03 ± 18.57 years, with similar gender distribution and fracture patterns between groups. Mean coronal HHS was lower in the cross-element group than in the conventional group, although the difference was not statistically significant (1.07 ± 2.29 mm versus 2.11 ± 2.64 mm, p=0.096). Axial HHS was similar between the groups (0.89 ± 3.93 mm versus 0.75 ± 3.34 mm, p=0.863).

Conclusion

The use of cross-elements in the PANTERA plate may reduce humeral head subsidence compared to a conventional plate system, potentially lowering the risk of screw penetration through the articular surface in proximal humerus fracture fixation.

## Introduction

Displaced fractures of the proximal humerus in adults are commonly treated conservatively, but surgical treatment remains a choice for complex fractures [1,2], particularly for high-energy injuries including head-split or Neer’s three- or four-part fractures, and fracture-dislocations [3,4].

For many years, angular stable locking plates have become popular for the fixation of proximal humerus fractures due to improved complication rates compared to conventional plates [4]. The PHILOS system is a commonly used locking plate system, although there are several similarly designed proximal humeral locking plates that aim to prevent varus collapse and articular screw perforation [5,6]. However, these complications remain issues, especially in patients with risk factors such as increasing age, osteoporosis, severe comminution, loss of medial cortical support, non-anatomic reduction and poor patient compliance [7-9]. Screw penetration is often associated with avascular necrosis of the humeral head or varus collapse, leading to secondary complications and subsequent reoperation [10,11]. Poor screw-bone interface is the primary mode of failure in the plate-screw construct [12], with joint reaction forces not being applied perpendicular to these locking screws in the metaphyseal segment.

In a recent systematic review, a high overall complication rate of 33% was reported in proximal humerus fractures fixed with various locking plate systems, including screw-related complications in 11% of patients [4]. Screw penetration represented 80% of all screw-related complications, and screw cut-out represented 10% overall. These complications were strongly associated with three- or four-part fractures in female patients over 50 years of age. Impaction of the humeral head frequently occurs during early fracture healing, which can lead to screw subsidence and ultimately penetration through the articular surface [13]. Impaction of up to 6.5mm has been reported in the presence of metaphyseal comminution treated with smooth wires or locking plates [14,15].

The PANTERA plate system is an innovative locking plate system that introduces "cross-element" (threaded screw) interlocking within the "locking posts" (larger locking screws) within the humeral head. This mechanism is designed to reduce the incidence of impaction by increasing the bone-implant interface, distributing the load vector across the larger surface area. The cross-elements create a three-dimensional scaffold that claims to help increase anchorage in osteoporotic bone and distribute the load orthogonally to the locking screws, keeping the load below the bone failure threshold [16].

A biomechanical study by Gonzalez-Hernandez et al. showed greater resistance to displacement and ability to sustain loads with a locking post/cross-element construct as compared to a conventional locking screw [16]. The addition of one or two cross-elements increased the peak load, work-to-peak load, and work resistance to further displacement significantly. However, these results have not been proved in clinical studies.

The aim of this study was to assess humeral head subsidence and screw penetration in proximal humerus fractures treated with cross-element fixation in comparison to conventional locking plates. We hypothesised that the use of cross-element fixation reduces the rate of humeral head subsidence and screw penetration compared to conventional locked plate fixation.

## Materials and methods

Patients with proximal humerus fractures who underwent open reduction internal fixation using a locking plate at a level one trauma centre between 2019 and 2023 were recruited in this retrospective study. The inclusion criteria involved widely displaced Neer’s two-, three-, or four-part fractures, which were not amenable to non-operative treatment [3]. Patients who underwent fixation with intramedullary devices, suture fixation, isolated screw fixation or arthroplasty were excluded. The study was initiated after obtaining approval from the Trust Clinical Effectiveness Unit (Reference 11775).

A total of 27 patients underwent proximal humerus fracture fixation with the PANTERA plate system during the study period. A total of 60 consecutive patients who had fixation using the PHILOS plate were identified from the operating theatre logbook and were used as the control (conventional group). Advanced age with osteoporosis, and initial fracture pattern with displacement are the two most important factors predicting outcomes after locking plate osteosynthesis of proximal humerus fractures [17]. The groups were matched as closely as possible. However, implant selection was not random and may have been influenced by surgeon preference and experience, implant availability, case timing and fracture complexity. These factors may have introduced selection bias and could have favoured either treatment group.

The surgeries were performed by two fellowship-trained consultant upper-limb surgeons or their fellows under direct supervision, using the deltopectoral approach in beach chair position. Anatomical reduction was achieved, and stabilisation of the fracture was performed with the PANTERA plate system using a published operative technique [16]. The tuberosities were secured to the plate clips (two anterior, one superior, and one posterior) using non-absorbable braided sutures, and cross-elements were inserted through the locking posts using a cross-element guide.

In the conventional group, stabilisation of the fracture was achieved with the PHILOS locking plate system using a published operative technique [18]. The number of locking screws used was based on the discretion of the operating surgeon, with a minimum of six proximal locking screws used in all cases. Intra-operative fluoroscopy was used to assess the reduction with coronal and axial views.

The long head of the biceps tendon was treated with either tenotomy or tenodesis towards the end of the operative procedure, within the rotator interval, to avoid damage to the ascending arcuate branch of the anterior circumflex humeral artery.

Post-operatively, the shoulder was immobilised in a broad arm sling for three weeks. Rehabilitation included physical therapy with passive range rehabilitation initially followed by active/assisted exercises beyond four weeks, and unrestricted activity with strengthening exercises beyond 12 weeks. All the patients were regularly reviewed in the follow-up clinic with radiographs until fracture union. Any complications or need for further surgery were recorded.

**Figure 1:**
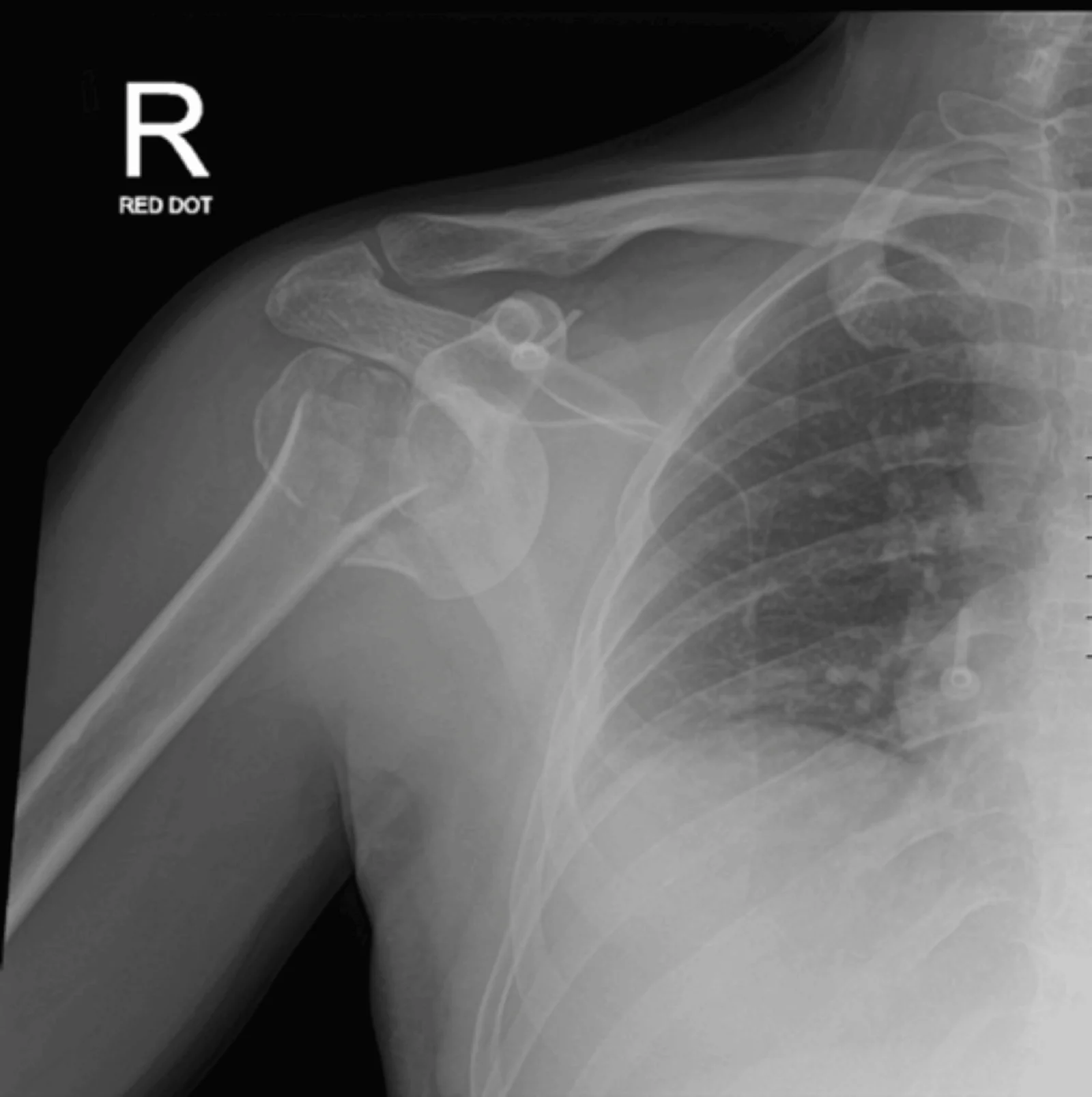
Pre-operative radiograph of a patient in the conventional group

**Figure 2:**
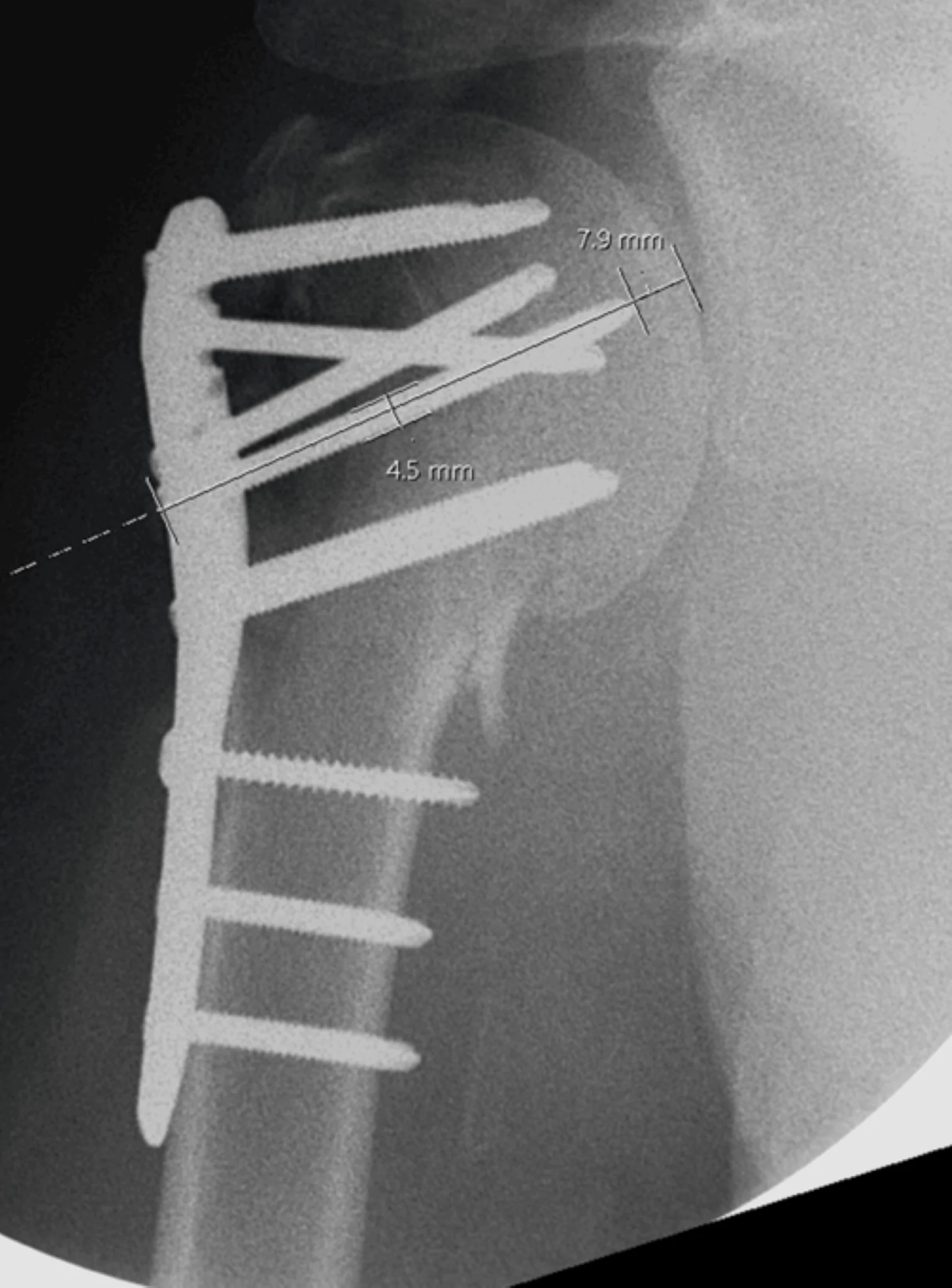
Intra-operative radiographs of the patient in the conventional group treated with the PHILOS plate Measurements of the intra-operative humeral head subsidence (HHS) from the screw tip to the articular surface = 7.9 mm. The locking screw coronal diameter (D_cor) = 4.5 mm.

**Figure 3:**
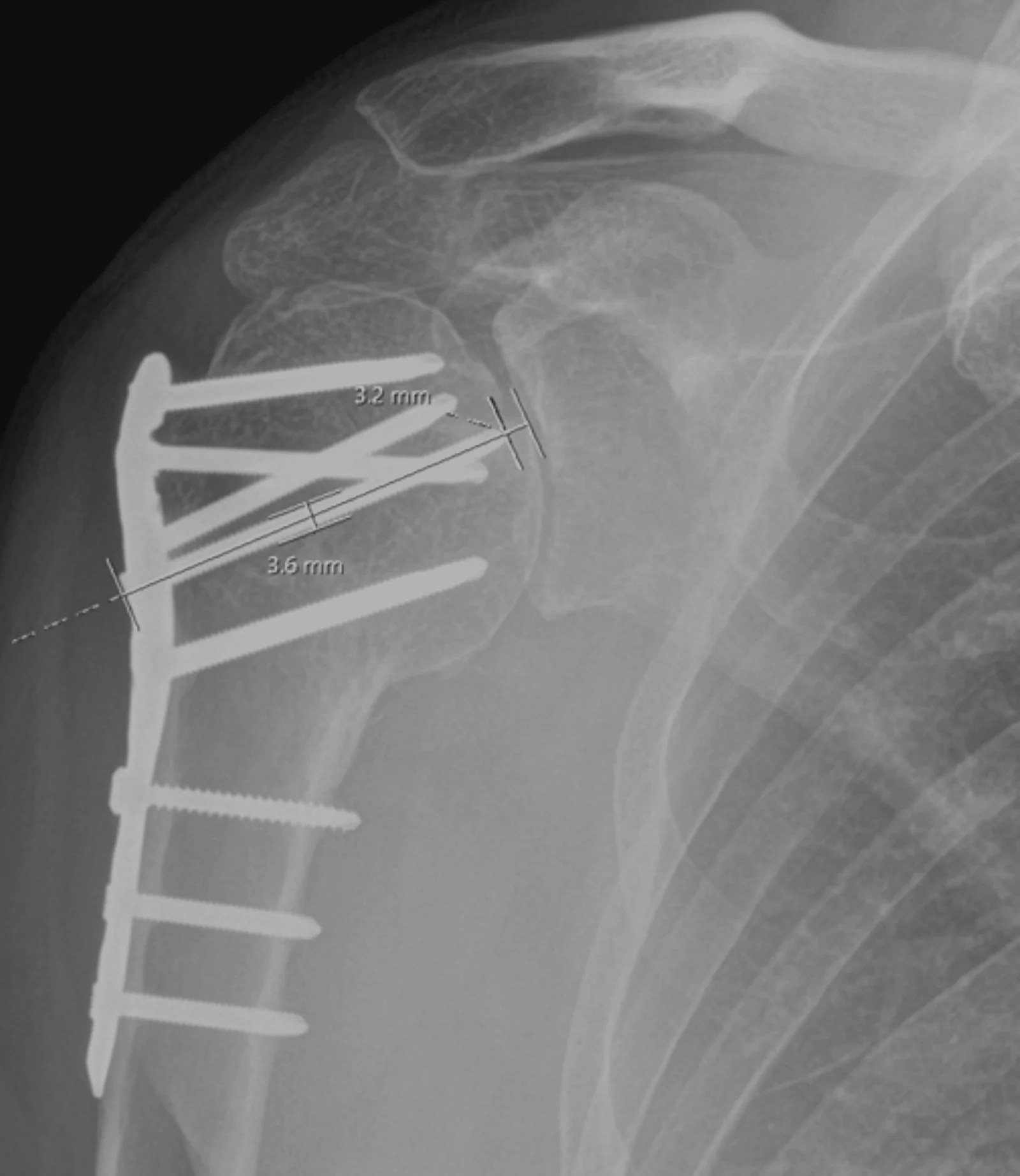
Post-operative radiograph of the patient in the conventional group demonstrating humeral head subsidence Measurements of the post-operative humeral head subsidence (HHS) from the screw tip to the articular surface = 3.2 mm. The locking screw cornoal diameter (D_cor) = 3.6 mm.

**Figure 4:**
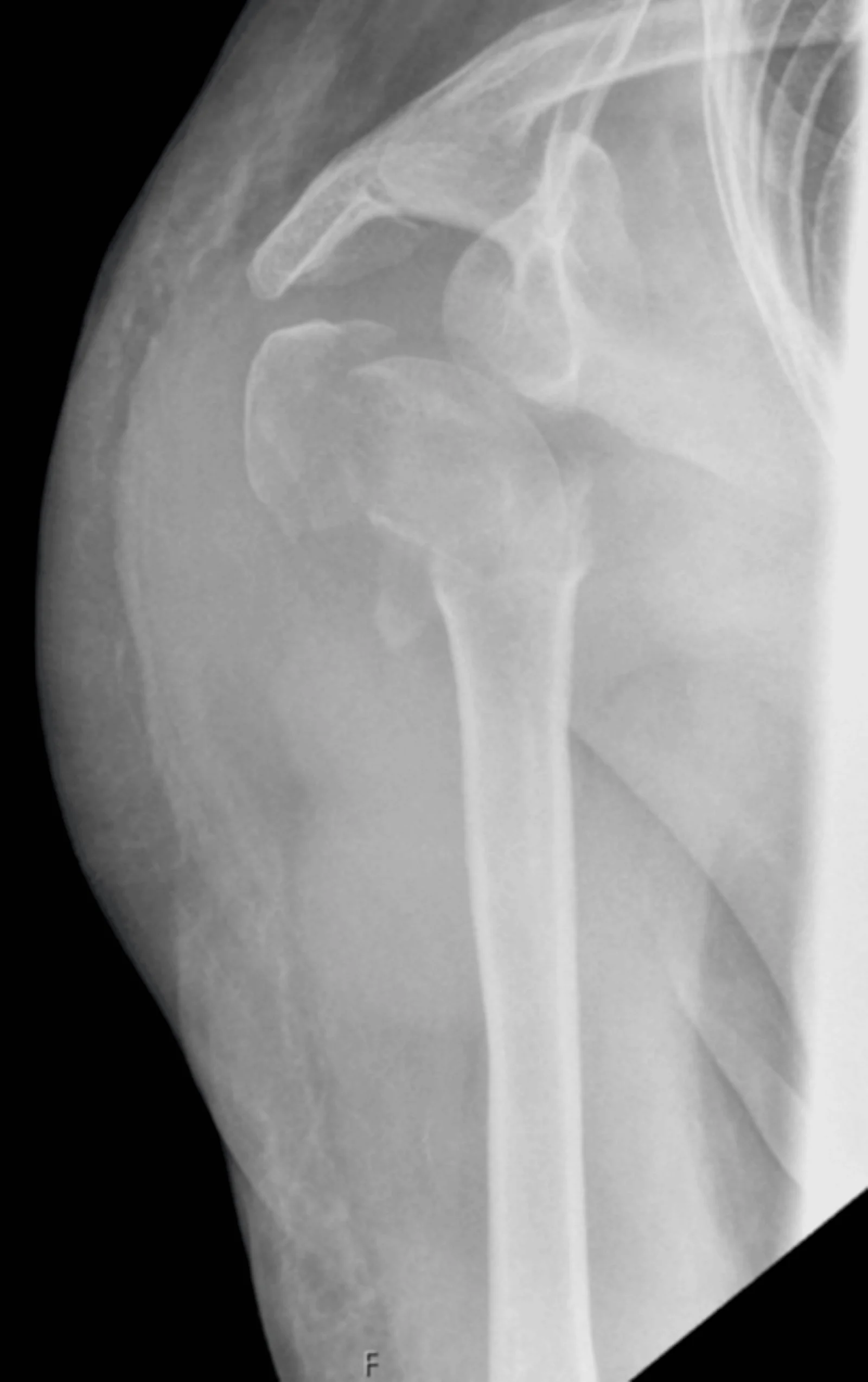
Pre-operative radiograph of a patient in the cross-element group

**Figure 5:**
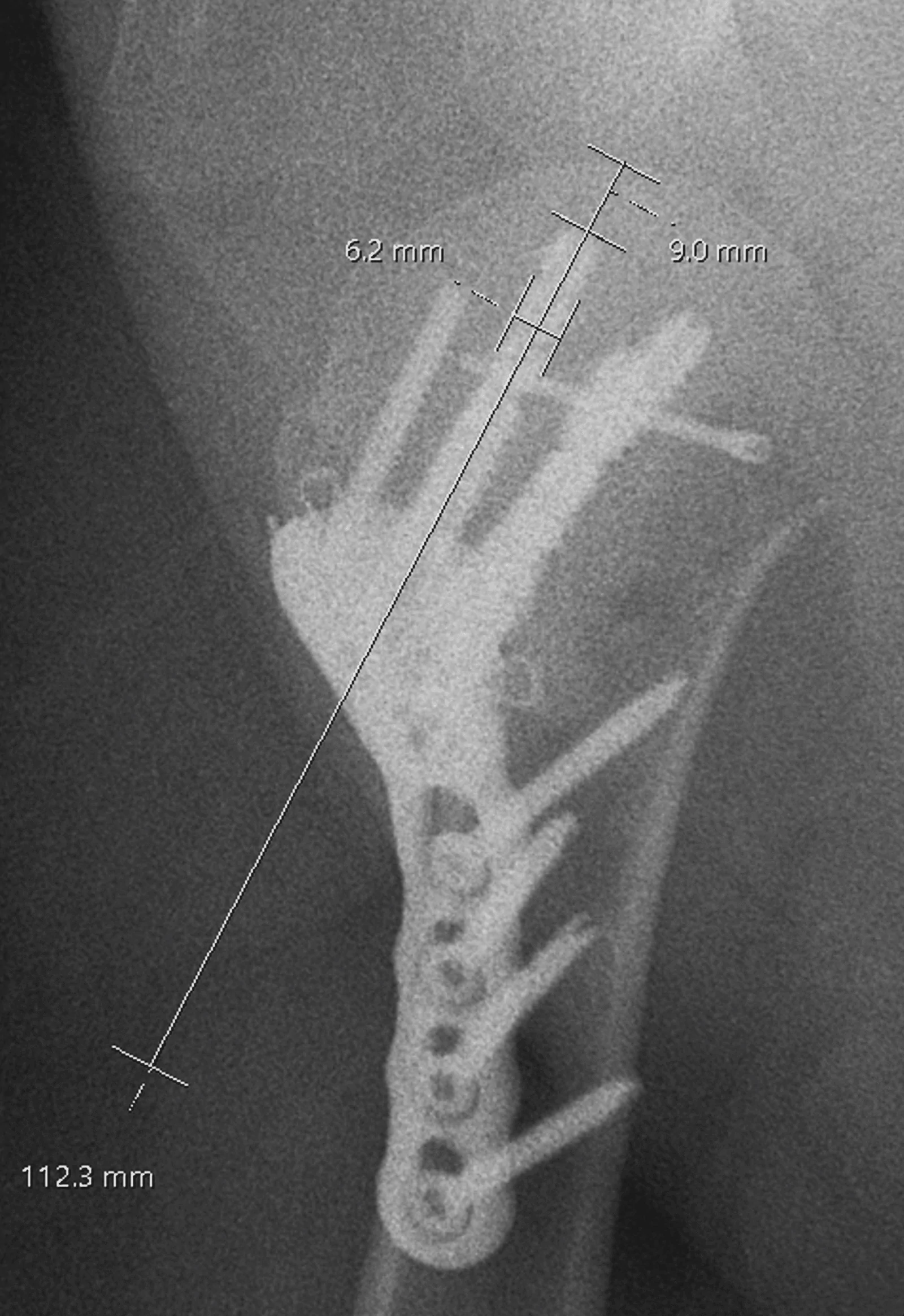
Intra-operative radiograph of the patient in the cross-element group treated with the PANTERA plate Measurements of the intra-operative humeral head subsidence (HHS) from the screw tip to the articular surface = 9 mm. The locking screw coronal diameter (D_cor) = 6.2 mm.

**Figure 6:**
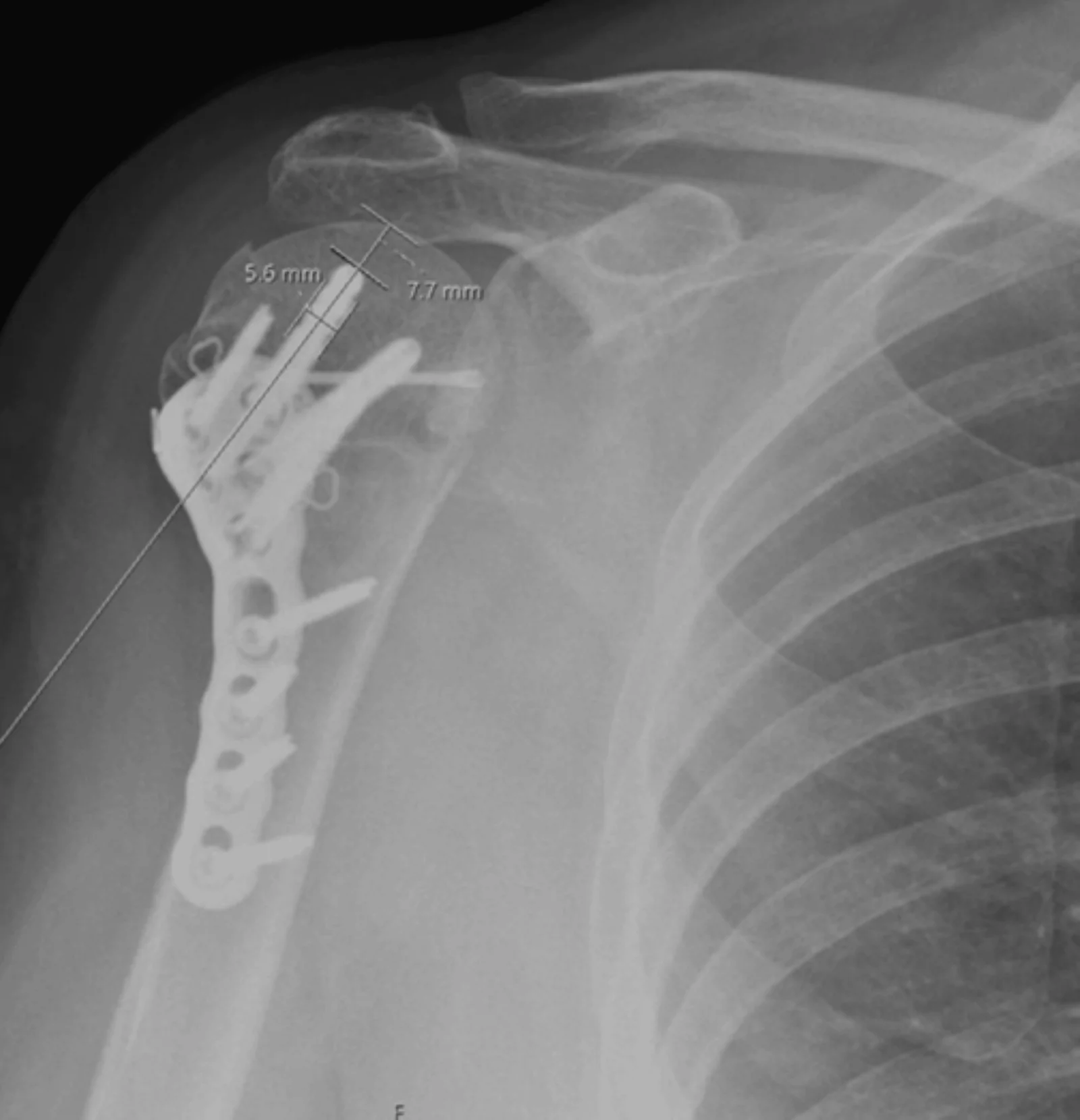
Post-operative radiograph of the patient in the cross-element group demonstrating HHS Measurements of the post-operative humeral head subsidence (HHS) from the screw tip to articular surface = 7.7 mm. The locking screw coronal diameter (D_cor) = 5.6 mm.

Figure 1 (pre-operative), Figure 2 (intra-operative) and Figure 3 (post-operative) radiographs demonstrate humeral head subsidence (HHS) in a patient in the conventional group with an intra-operative coronal screw-head distance of 7.9 mm, post-operative coronal screw-head distance of 3.2 mm, and Cor-HHS = 4.7 mm. Figure 4 (pre-operative), Figure 5 (intra-operative), and Figure 6 (post-operative) radiographs for the PANTERA plate demonstrate HHS in a patient in the cross-element group with an intra-operative coronal screw-head distance of 9 mm, post-operative axial screw-head distance of 7.7 mm, and Ax-HHS = 1.3 mm. To quantify the impaction, the radiographic index of humeral head subsidence was compared using serial radiographs of the same view, and distance was measured along the axis line of the screw, from the tip of the screw to the articular surface. Correction of radiographic magnification was performed by calculations comparing the apparent diameter of the proximal locking screw with its known size, as shown in Figure 2 and Figure 3 for a patient from the conventional group and Figure 5 and Figure 6 for a patient from the cross-element group.

For the conventional group, the magnification was calculated by using the known diameter of the locking screw (D_true; e.g. 3.5 mm) to the apparent diameter on the radiograph (D_cor on the coronal X-ray, and D_ax on the axial X-ray). Similarly, for the cross element group, the magnification was calculated by using the known diameter of the locking screw (D_true; e.g. 5.2 mm) to the apparent diameter on the radiograph (D_cor on the coronal X-ray, and D_ax on the axial X-ray).

$Cor HHS= ( Distance _{postop} x \frac{D_true}{D_cor} )-( Distance_{ intraop} x \frac{D_true}{D_cor} )$

This was measured on intra-operative fluoroscopy and final post-operative radiographs at the routine clinic follow-up. Subsequently, HHS was calculated as the difference in screw tip to articular surface distance between the two radiographs (post-operative - intra-operative) for both coronal (Cor-HHS) and axial (Ax-HHS) views.

Two independent assessors evaluated the radiographs on two different occasions at least 15 days apart. The radiographic measurements were performed electronically on Carestream Vue Patient Archiving and Communication System (PACS) (Carestream Health, Inc., 2020, Rochester, NY) using image analysis software.

Statistical analysis

Statistical analysis was performed using SPSS 22.0 (IBM Corp, Armonk, NY, USA). Continuous variables were presented as mean±SD and categorical values were presented as numbers or percentages. Intra- and inter-observer reliability was assessed using the Bland-Altman method for agreement between the two assessors [19]. The distribution of pre-operative characteristics between the two groups was compared using Student's t-test for quantitative variables and the chi-square test/Fisher exact test for qualitative variables. The radiographic measurements were compared between the two groups using Student's t-test. The complications were compared between the two groups using the chi-square test/Fisher exact test, with a p-value less than 0.05 considered statistically significant.

## Results

The mean age of patients in both groups was 55.03±18.57 years (range, 21 to 85 years), with a male predominance in both groups (35 out of 60 (58.33%) and 16 out of 27 (59.26%) in the conventional and cross-element groups, respectively). The two groups were similar in their pre-operative characteristics; follow-up duration was similar in both groups, with an average of 21.16±22.94 weeks for the conventional group and 24.24±13.40 weeks for the cross-element group. There was no loss to follow-up. The descriptive statistics are presented in Table 1.

**Table 1:** Descriptive statistics This table shows the total number of patients, gender, side of fracture and Neer classification [3] for both the PHILOS and PANTERA groups.

Variables	PHILOS	PANTERA
n	60	27
Male	35	16
Female	25	11
Right	28	14
Left	32	13
Neer Class 2	28	5
Neer Class 3	24	15
Neer Class 4	8	7

The interobserver reliability between the two assessors was excellent, with minimal bias of 0.002 (SD 0.014) (limit of agreement -0.025 to +0.03) for Cor-HHS and -0.002 (SD 0.021) (limit of agreement -0.043 to +0.04) for Ax-HHS. Intra-observer reliability showed minimal bias of 0.002 (SD 0.018) (limit of agreement -0.033 to +0.037) for Cor-HHS and 0.002 (SD 0.012) (limit of agreement -0.021 to 0.025) for Ax-HHS.

**Figure 7:**
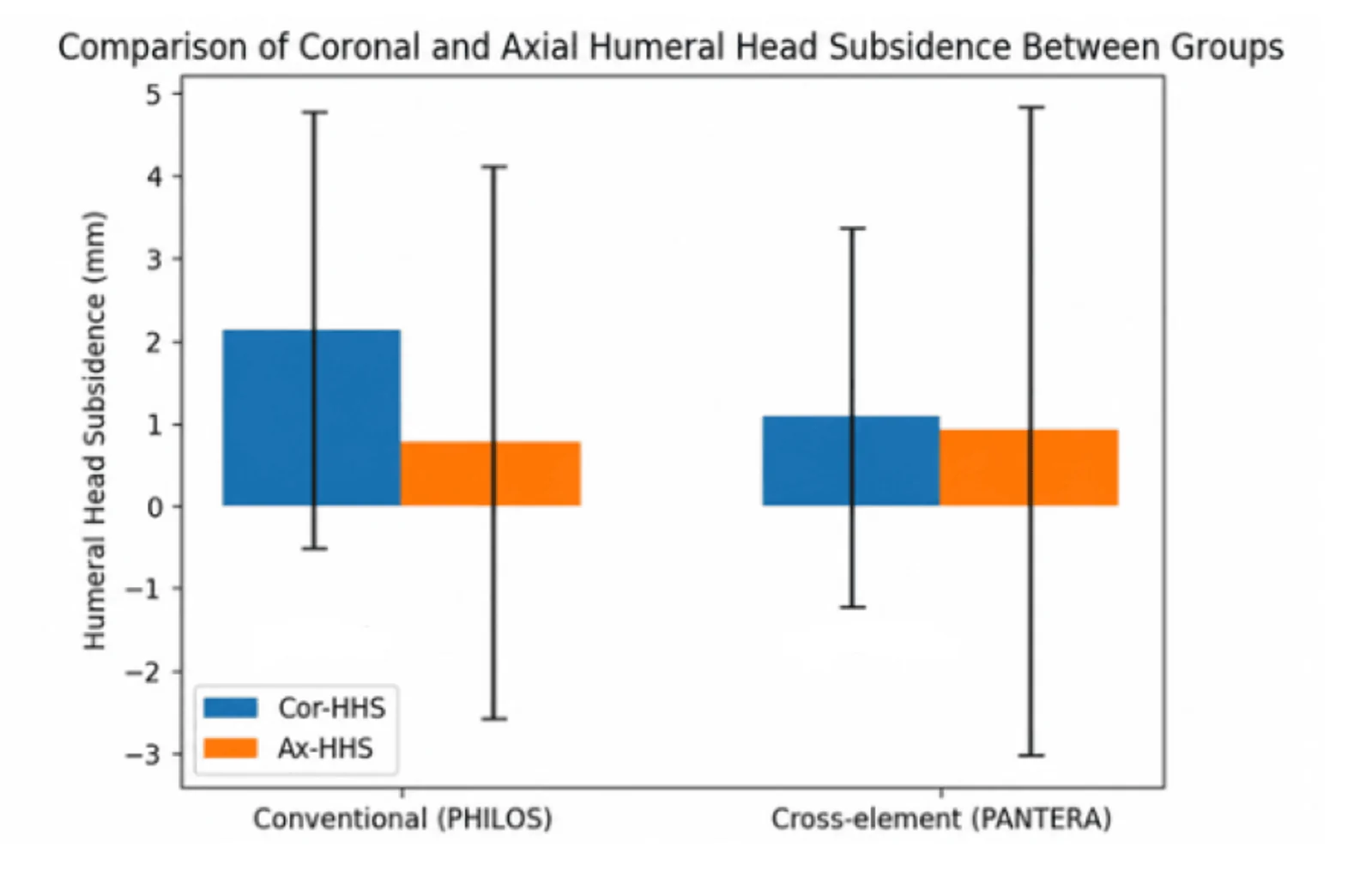
Comparison of coronal and axial humeral head subsidence between groups

None of the patients in both groups had screw penetration at the time of surgery (intra-operatively). At the point of final follow-up, 20 (33.33%) patients in the conventional group were noted to have screw subsidence with an increase in screw-to-head height (both coronal and axial) on the final post-operative radiograph, compared to 1 (3.70%) patient in the cross-element group (p=0.45). Mean coronal HHS was lower in the cross-element group than in the conventional group, although the difference did not reach statistical significance (1.07 ± 2.29 mm versus 2.11 ± 2.64 mm, p=0.096). Mean axial HHS was similar between the groups (0.89 ± 3.93 mm versus 0.75 ± 3.34 mm, p=0.863) (Figure 7 and Table 2). 

**Table 2:** Humeral head subsidence results comparison of the PHILOS and PANTERA groups

Parameter	PHILOS	PANTERA	p-value
Cor-HHS (mm)	2.11 ± 2.64	1.07 ± 2.29	0.096
Ax-HHS (mm)	0.75 ± 3.34	0.89 ± 3.93	0.863

Four patients in the conventional group experienced HHS and screw penetration, had elements of avascular necrosis and required further surgery to rectify this. Another three patients suffered from adhesive capsulitis that required non-operative treatment. One patient developed heterotopic ossification and was managed conservatively.

In the cross-element group, one patient had a screw cut-out and loss of reduction, with possible avascular necrosis, and was revised to a reverse-polarity shoulder replacement. Three patients suffered from adhesive capsulitis, and one patient developed heterotopic ossification, all of them treated successfully with conservative treatment.

## Discussion

Our study demonstrated lower mean coronal humeral-head subsidence with cross-element fixation; however, the difference was not statistically significant. Axial subsidence was similar between the groups. Screw penetration was less frequently observed in the cross-element group, but this difference was also not statistically significant and should be interpreted cautiously because of the limited sample size and statistical power.

In the conventional group, screw subsidence with the use of the locking plate system was consistent with previous studies. Owsley et al. reported more than 3 mm screw subsidence in 23% of patients treated with proximal humeral locking plates, with all resulting in screw penetration through the articular surface [15]. In the current study, in the cross-element group, the HHS value was 1.06 mm and 0.89 mm in both antero-posterior (AP) and axial radiographs between the intra-operative and final post-operative images, indicating more successful prevention of screw subsidence and humeral head impaction.

Screw penetration, which occurred in a significant proportion of patients in the conventional group, is a recognised complication associated with locking plates [20,21]. Kralinger et al. observed screw penetration in 34 (22.7%) patients in a prospective analysis of 150 patients treated with the PHILOS plate [20]. Similarly, Beeres et al. retrospectively analysed 282 patients treated with a locking plate and noted screw penetration in 23% of patients [21]. In contrast, screw penetration was rarely observed in the cross-element group; although this difference did not reach statistical significance, it suggests the potential positive impact of cross-elements.

The cross-elements are designed to counteract the axially directed joint reaction force in the glenohumeral joint by providing resistance perpendicular to the joint reaction force vector. This principle is similar to that of internal fixation in other bones, such as the distal radius or tibial plateau, where locking screws are oriented parallel to the joint surface, providing enhanced resistance to joint reaction forces [22]. Emerging evidence suggests that the blunt tip, larger core diameter, and lower thread pitch of screws have a positive effect on reducing screw penetration and increasing construct stability [23,24]. The 5.2 mm post used with the PANTERA plate system has a larger core diameter and blunt ends, which may have contributed to reducing screw subsidence compared to conventional plates that use locking screws with a smaller core diameter (3.5 mm) and pointed tip.

Previous studies have used the distance from the screw tip to the articular surface to measure the amount of head impaction or screw subsidence [6,15]. However, this method has limitations, as it may not be accurate on non-calibrated fluoroscopic images. To overcome this limitation, Carbone et al. measured the distance from the K-wire tip to the articular surface on fluoroscopic images and post-operative radiographs to assess the amount of impaction in fractures treated with Humerusblock devices [14]. However, their technique does not apply to other implant devices such as locking plates. In our study, we measured the calibrated HHS along a fixed point - the screw axis - which was reproducible in radiographs taken at different times. The Bland-Altman analysis supported the internal validity of this calculation. Although a CT scan would have been a better mode of investigation to measure HHS at both immediate post-operative and final follow-up, it was not feasible in our public hospital setting due to concerns about radiation exposure and costs.

Avascular necrosis of the humeral head is a commonly encountered complication, with a reported incidence of up to 33% in patients undergoing surgical fixation [10]. Risk factors for avascular necrosis include fracture configuration, length of metaphyseal head extension, integrity of the medial hinge, and fracture patterns involving displaced head fragments [25,26]. In our study, avascular necrosis was seen in four patients in the conventional group, while only one occurred in the cross-element group. We postulate that more stable fixation of the lesser tuberosity to the humeral head is achieved with cross-elements, which helps in revascularisation of the humeral head. Most of the humeral head is supplied via the arcuate branch of the anterior circumflex humeral artery at the proximal portion of the bicipital groove [27], which forms part of the lesser tuberosity fragment in three- or four-part fractures [28].

The group matching and nil loss to follow-up were strengths of this study. A major limitation of this study was its retrospective nature; however, group matching was undertaken to reduce the bias related to major confounding factors, such as age and fracture configuration [29-31], which are known to negatively affect screw penetration [4]. Another significant limitation was the small sample size, which might have reduced the ability to detect significant differences in screw penetration rate between the two groups. Furthermore, a longer follow-up period would be ideal; however, this is unlikely to affect the outcome of the study to detect the screw penetration rate, as all the patients were followed up until radiological fracture healing, beyond which further screw subsidence and penetration is unlikely to happen. This short length of time of follow-up may reflect the lower incidence of complications seen or perhaps better outcomes in the cross-element group; however, this was not evaluated in this study. Finally, the sample size to demonstrate 80% power was not prospectively calculated. However, the post-hoc power analysis with an alpha error of 0.05 revealed 89.9% power for the detection of screw subsidence and 60% power for screw penetration. Implant allocation was not random and may have been influenced by surgeon experience, implant availability, case timing and fracture complexity. Consequently, unmeasured differences between the groups may have influenced the observed outcomes.

Although it has been proven biomechanically that increasing the number of cross-elements increases the screw pull-out characteristics [16], the minimum number of cross-elements recommended to improve the clinical outcome is currently undetermined. In the authors’ experience, using at least one cross-element was as effective as using two or three cross-elements; however, the small number of patients restricts the widespread applicability of such a conclusion.

## Conclusions

Lower coronal humeral-head subsidence was observed with cross-element fixation; however, the differences in coronal and axial subsidence between the groups were not statistically significant. The study had 89.9% post-hoc power for assessing subsidence but only 60% power for screw penetration; therefore, the lower observed screw-penetration rate in the cross-element group should not be interpreted as a confirmed protective effect. A sufficiently powered randomised controlled trial is required to determine whether cross-elements reduce humeral-head subsidence and screw penetration following proximal humerus fracture fixation.
